# Cost-Trajectory Framework for Total Episode Expenditure in Complex Wound Reconstruction: The CASCADE (Cost Analysis of Surgical Complications and Downstream Expenditure) Model

**DOI:** 10.7759/cureus.108363

**Published:** 2026-05-06

**Authors:** Andrew M Klapper, Anthony N Dardano, Michael Risin, Karla Maita

**Affiliations:** 1 Plastic and Reconstructive Surgery, Delray Medical Center, Delray Beach, USA

**Keywords:** cost analysis, healthcare economics, orthoplastic surgery, reconstructive surgery, surgical site infection, value-based care, wound reconstruction

## Abstract

Complex wound reconstruction may progress through stage-dependent clinical and economic trajectories in which wound failure leads to infection, reoperation, prolonged hospitalization, post-acute care, outpatient wound management, and possible readmission. Procedural-cost evaluation may not fully capture these downstream consequences in high-risk reconstructive settings. This paper presents the CASCADE (Cost Analysis of Surgical Complications and Downstream Expenditure) model, a conceptual, literature-derived decision framework rather than a primary data analysis or statistically validated predictive model. CASCADE applies an expected-value framework to evaluate the reconstructive strategy at the index operation, using three bounded inputs: failure probability (P), failure trajectory cost (C), and incremental reconstruction cost (ΔC). These inputs are structured into the decision rule ΔC < ΔP × C. A Clinical Risk Score (CRS) is used to standardize the assignment of cases into risk tiers. Across illustrative high-risk wound environments, failure trajectories may increase total episode expenditure from approximately $80,000-$150,000 after successful reconstruction to $400,000-$1,000,000+ after failure-driven care. These values are literature-informed illustrative estimates used to parameterize the framework, not observed case-level measurements. Sensitivity analysis demonstrates that at CRS ≥6, the CASCADE decision threshold often exceeds typical incremental reconstructive procedure costs across plausible input combinations. CASCADE provides a reproducible, bidirectional framework for trajectory-based cost evaluation in complex wound reconstruction. It may support surgical decision-making, institutional planning, and reimbursement analysis when total episode cost, rather than index procedural cost alone, is the appropriate unit of economic evaluation. The framework evaluates cost relationships but does not define reimbursement levels and requires future validation against institutional or claims-based datasets.

## Introduction and background

Complex wound reconstruction presents a fundamental systems question: will the episode remain finite, or will it progress into a prolonged failure trajectory? In this manuscript, stage-dependent cost progression refers to the sequence by which wound failure may lead to infection, reoperation, prolonged hospitalization, post-acute care, outpatient wound management, and possible readmission. This trajectory is influenced by the index operation, where the initial reconstructive strategy establishes biological and mechanical conditions that affect the probability and course of subsequent outcomes. Once complications such as surgical site infection (SSI) occur, the episode may transition into a resource-intensive state characterized by increased utilization, cost, and morbidity, as consistently demonstrated in large cohort and population-based analyses [1,2].

In high-risk wound environments, including posterior spine surgery, oncologic resection, trauma reconstruction, and diabetic limb salvage, wound failure is often associated with recognizable clinical patterns. Initial technical or biological compromise may lead to infection, reoperation, prolonged hospitalization, post-acute care, and functional decline. Each stage may alter the wound environment, constrain subsequent intervention, and increase the probability and cost of further deterioration [3-6].

Within this framework, failure is not treated as a fully predictable individual outcome. Rather, CASCADE (Cost Analysis of Surgical Complications and Downstream Expenditure) evaluates wound failure as a probabilistic event whose risk, clinical pathway, and cost consequences may be estimable within defined high-risk populations. The distinction matters for economic evaluation: an individual complication may be uncertain, but complication rates, downstream treatment patterns, and episode-level cost ranges can be evaluated across comparable clinical settings. CASCADE is structured around this population-level, probability-based distinction [1-4].

Despite this, many economic evaluations remain anchored to procedural cost at a single time point. This may create a structural mismatch: decisions are assessed at the index procedure while consequences unfold across a multi-stage clinical trajectory spanning inpatient, post-acute, and outpatient domains. In high-risk cases, this approach may underestimate the total episode expenditure associated with wound failure and may fail to capture the value of definitive reconstructive strategies [3,4].

The CASCADE model addresses this gap by shifting the unit of analysis from isolated procedural cost to total episode expenditure, incorporating complication probability, downstream resource utilization, and trajectory-dependent cost escalation. The framework is designed for prospective application at the index operation and for retrospective evaluation in reimbursement and value-based care contexts. CASCADE is a conceptual, literature-derived framework. It is not a primary data analysis, a claims-based economic study, or a statistically validated patient-level prediction tool. Its purpose is to organize established risk factors and plausible downstream treatment trajectories into a transparent expected-value structure that can be tested in future empirical studies.

In multi-stage reconstructive pathways, any evaluation limited to the index procedure necessarily omits the majority of cost-generating events when failure occurs. The resulting determination is not merely incomplete; it evaluates a different economic unit than the one actually put at risk by the operative decision. This study presents a conceptual, literature-derived framework rather than a data-driven or empirically validated model.

Foundational principle

CASCADE does not introduce new variables or define pricing. It reorganizes independently documented risk factors and cost consequences into an expected-value framework for evaluating total episode expenditure. Each input domain corresponds to established clinical evidence; no inputs are self-generated or institution-specific.

## Review

Methods

Unit of Analysis

For multi-stage clinical pathways, the appropriate economic unit is total episode cost conditional on index strategy selection, rather than the cost of the index procedure alone. Because downstream events are conditional on the index strategy, the total episode cost is path-dependent and cannot be fully evaluated at a single time point.

Failure-related costs may extend across the care continuum, generating costs at each transition point that are difficult to attribute to the index decision when evaluated at a single time point (Table 1).

**Table 1:** Distribution of Failure-Related Costs Across Care Settings and Limitations of Attribution to Index Surgical Decision-Making Failure-related costs extend beyond the index hospitalization and are distributed across multiple care settings and financial domains. These costs are frequently fragmented, delayed, or excluded from procedural accounting, which may contribute to incomplete estimation of the economic impact of failure trajectories in reconstructive surgery.

Care Setting	Failure-Driven Impact	Cost Attribution Limitation
Inpatient	Extended length of stay; additional OR utilization; ICU-level care; specialist consultation	Costs accrue after the index procedure and are not attributable to the initial operative decision
Post-Acute	SNF placement; home health; wound care nursing; IV antibiotics	Fragmented billing across separate episodes; frequently attributed to patient factors rather than index strategy
Outpatient	Repeated clinic visits; wound care supplies; physical therapy; prolonged follow-up care	Distributed across multiple encounters; rarely aggregated into total episode cost
Institutional	Readmission penalties; bundled payment overruns; OR throughput disruption; quality metric deterioration	Systematically excluded from per-case procedural accounting and surgeon-level valuation

This diffusion of cost across settings and billing episodes is not incidental - it is structural. Evaluation frameworks anchored to the index procedure cannot capture costs that, by definition, occur downstream of it. CASCADE aggregates these domains into a single expected-value calculation, making the full economic unit visible at the point of decision.

CASCADE defines two mutually exclusive index strategies: S₁ (routine closure) and S₂ (definitive reconstruction). Expected total episode cost under each is E[Cost | S₁] = C₀ + P₁ × C and E[Cost | S₂] = C₀ + ΔC + P₂ × C. S₂ is favored when ΔC < (P₁ − P₂) × C = ΔP × C, where C₀ = baseline episode cost (constant; cancels from comparison); P₁ = failure probability under routine closure; P₂ = failure probability under reconstruction; ΔC = incremental reconstruction cost (facility/operative; physician fees excluded); and C = expected failure trajectory cost conditional on failure.

The model intentionally compares two simplified index strategies, routine closure and definitive reconstruction, to demonstrate the expected-value relationship. In practice, intermediate or hybrid strategies may be used, including staged debridement, temporary negative-pressure therapy, delayed closure, local tissue rearrangement, regional flap coverage, or escalation to free-tissue transfer. These clinical variations can be incorporated by adjusting ΔC, P₂, and expected failure trajectory cost according to the strategy being evaluated.

Step 1 - Clinical Risk Score (CRS)

Risk factors are scored additively. The CRS provides a standardized basis for input assignment, translating clinical features into bounded failure probability ranges (Table 2). Scoring reflects cumulative risk burden; risk factors frequently coexist in high-risk wounds (Table 3). The CRS is a nonvalidated, non-statistically derived clinical stratification tool intended to standardize input assignment. Two-point factors were assigned to wound- or reconstruction-specific features that directly affect mechanical stability, perfusion, implant exposure risk, or prior failure biology. One-point factors were assigned to broader contextual or systemic risk amplifiers that increase complication probability but may be less determinative when present in isolation. This weighting is expert-informed and literature-supported rather than formally derived from regression modeling. Future refinement should test whether regression-based weighting or consensus methods, such as Delphi methodology, improve calibration and reproducibility.

**Table 2:** Clinical Risk Score (CRS) Components and Point Assignment CRS = Clinical Risk Score; total score range 0–12, with higher scores indicating greater risk of reconstructive failure.

Risk Factor	Criteria	Points
Perfusion compromise	Ischemia, radiation history, tenuous tissue	2
Hardware present	Instrumentation, prosthetics, implants	2
Closure tension	Inability to achieve tension-free closure	2
Soft tissue destruction	Dead space, muscle loss, tissue deficit	2
Prior failure	Prior wound breakdown or revision	2
Contamination	Non-sterile field, colonization, open wound	1
Operative factors	Prolonged surgery, reoperation field	1
Comorbidity	Diabetes, obesity, immunosuppression, malnutrition	1

**Table 3:** Clinical Risk Score (CRS) Risk Tier Stratification CRS = Clinical Risk Score; risk tiers stratify the likelihood of reconstructive failure based on the cumulative score.

Cumulative CRS	Risk Tier
0–2	Low
3–5	Moderate
6–8	High
9+	Extreme

The CRS uses additive scoring for practical application and transparency. This approach does not model interaction effects between variables, such as the combined effect of diabetes, contamination, and hardware exposure. Such interactions may be clinically important and should be evaluated in future validation studies using multivariable or probabilistic modeling.

Step 2 - Failure Probability Assignment

Failure probabilities are derived from published wound complication rates stratified by risk environment (Table 4). Ranges reflect the convergence of independently documented risk amplifiers (perfusion deficit, hardware presence, closure tension, contamination). Use the midpoint for typical cases; upper bound for clustered high-risk features; lower bound if borderline. All parameter ranges represent bounded estimates derived from convergent findings across multiple independent studies rather than single-source inputs [5-8].

**Table 4:** Estimated Failure Probability by Risk Tier and Impact of Reconstruction P₁ = estimated probability of failure with routine closure; P₂ = estimated probability of failure with reconstructive strategy; ΔP = absolute risk reduction. Values represent literature-informed ranges across high-risk wound settings.

Risk Tier	Routine Closure (P₁)	Reconstruction (P₂)	ΔP (Absolute Risk Reduction)
Low	0.05–0.10	0.02–0.05	0.03–0.05
Moderate	0.10–0.15	0.03–0.07	0.05–0.10
High	0.15–0.25	0.05–0.10	0.10–0.15
Extreme	0.25–0.40	0.07-0.15	0.15–0.25

The CRS is a structured aggregation of established risk factors rather than a statistically derived scoring system. Each domain corresponds to independently documented risk amplifiers with published associations to wound complication rates; scores are additive and reflect cumulative risk burden rather than isolated variable effects.

Parameter ranges were selected from the literature addressing SSI costs, complex wound failure, flap reconstruction, high-risk spine wounds, orthoplastic trauma reconstruction, oncologic perineal reconstruction, and diabetic or immunocompromised wound populations. The ranges were not generated from a formal meta-analysis. Instead, they represent bounded estimates derived from convergent findings across clinically related literature. When published estimates varied, ranges were selected to preserve conservative directional interpretation rather than to imply precise prediction.

Step 3 - Failure Cost Tier (FCT)

Select the tier reflecting the expected cost trajectory if failure occurs (Table 5). Selection is based on the likely progression given wound environment and anatomy, not best-case assumptions. Cost ranges reflect aggregate episode-level resource utilization (facility, anesthesia, perioperative, post-acute) and are calibrated to published SSI and wound failure cost literature [1-4]. Cost ranges are presented as literature-informed approximate estimates rather than inflation-adjusted or geography-specific values. They are intended to represent broad order-of-magnitude episode-level expenditure across complex wound settings. Actual costs may vary by year, geography, payer mix, hospital cost structure, post-acute utilization, and case complexity.

**Table 5:** Failure Trajectory Scenarios and Associated Total Episode Cost Ranges Cost ranges represent total episode expenditure, including downstream complications, reoperations, post-acute care, and institutional costs. Values are literature-informed estimates across complex wound settings. SSI = Surgical Site Infection; LOS = Length of Stay; VAC = Vacuum-Assisted Closure; SNF = Skilled Nursing Facility

Tier	Scenario	Clinical Events	Cost Range
1	Limited failure	SSI, minor reoperation, prolonged LOS	$100,000–$250,000
2	Moderate failure trajectory	Washout, VAC therapy, delayed closure	$250,000–$400,000
3	Severe failure trajectory	Multiple reoperations, readmission, SNF	$400,000–$800,000
4	Catastrophic failure trajectory	Free flap salvage, prolonged hospitalization, rehabilitation	$800,000–$1,000,000+

Steps 4-5 - Incremental Cost Input and Decision Execution

ΔC (incremental reconstruction cost) is case-dependent and externally determined by operative complexity, institution, and payer structure. In practice, ΔC may be estimated using institutional facility cost accounting, operating room time, implant or supply costs, anesthesia and perioperative resource use, diagnosis-related group (DRG)-based facility estimates, or payer-specific allowed amounts when available. Physician professional fees are excluded from CASCADE analysis, as they do not influence failure trajectory structure or expected-value relationships; inclusion would proportionally affect both strategies without altering directional conclusions.

Apply the decision rule: if ΔC < ΔP × C, reconstruction is favored on an expected total episode cost basis. The preferred strategy is the one that minimizes expected total episode expenditure, not the one with the lowest index procedural cost.

Model Scope and Deliberate Constraints

CASCADE is a decision-support and evaluative framework (Table 6). It does not establish reimbursement benchmarks, define procedural pricing standards, or assign physician reimbursement levels. Physician professional fees are excluded from the model, as they do not alter failure trajectory structure or expected-value relationships; inclusion would proportionally affect both strategies without changing directional conclusions. The model evaluates whether a strategy reduces expected total episode expenditure, independent of payer-specific rates or contractual pricing structures. Its inputs are derived from published complication rates and cost ranges; its conclusions reflect underlying cost dynamics rather than pricing assumptions. CASCADE evaluates economic relationships; it does not determine fee validity.

**Table 6:** Model Constraints and Scope Definitions

Constraint	Scope	Rationale
Physician professional fees excluded	Not modeled in either strategy	Professional fees do not alter failure trajectory structure or directional conclusions
No reimbursement benchmarks incorporated	Model evaluates cost relationships only	Separates trajectory-based cost analysis from fee determination
Inputs from published literature	Complication rates and cost ranges	No self-generated or institution-specific inputs used
Payer-structure independent	Conclusions apply across commercial, government, and self-pay	Reflects underlying cost dynamics rather than contractual pricing

Non-Applicability Criteria

CASCADE analysis is designed for high-risk wound environments where failure trajectory entry is clinically meaningful. The model does not apply uniformly across all wound presentations.

CASCADE analysis may not be applicable in the following settings: CRS 0-2 (low-risk wound environment); reliable tension-free closure achievable without advanced technique; no contamination, hardware, or perfusion compromise present; or standard comorbidity burden with no additional risk amplifiers. In such cases, routine closure may represent the clinically and economically appropriate strategy, and CASCADE analysis does not support a different conclusion.

Results

Two-Way Sensitivity Analysis: Decision Threshold by ΔP and FCT

Table 7 shows the maximum ΔC at which reconstruction remains favored (ΔP × C) across the full range of ΔP values and FCTs. Reconstruction is favored whenever ΔC falls below the corresponding cell value. Thresholds exceeding $50,000 indicate values within or above the range in which many incremental reconstructive procedure costs may fall.

**Table 7:** Failure Cost Thresholds (C × ΔP) by Risk Tier and Probability Reduction Values represent the failure cost threshold (C × ΔP), indicating the maximum incremental reconstruction cost justified by a given reduction in failure probability. Tier values reflect representative total episode costs from Table 5. Table 7 uses representative tier values to demonstrate the decision threshold relationship. It is a deterministic sensitivity analysis, not a probabilistic simulation. Because actual failure costs may vary across the full range of each tier, threshold values should be interpreted as illustrative decision boundaries rather than precise forecasts. Future validation should evaluate lower- and upper-bound thresholds and may incorporate Monte Carlo simulation across cost and probability distributions.

Failure Cost Threshold (C × ΔP)	ΔP = 0.05	ΔP = 0.10	ΔP = 0.13	ΔP = 0.20	ΔP = 0.25
Tier 1 ($175,000)	$8750	$17500	$22750	$35000	$43750
Tier 2 ($325,000)	$16250	$32500	$42250	$65,000	$81,250
Tier 3 ($600,000)	$30000	$60,000	$78,000	$120,000	$150,000
Tier 4 ($900,000)	$45000	$90,000	$117,000	$180,000	$225,000

Directional robustness: Across all high-risk and extreme-risk tier combinations (CRS ≥6), the decision threshold exceeds $50,000 for every ΔP value at or above 0.10 (Table 8). This means that, in clinical settings where CASCADE analysis is indicated, reconstruction is favored on expected-value grounds across the full realistic range of reconstructive procedure costs, without requiring favorable input selection.

**Table 8:** CASCADE Decision Rule Interpretation Based on Cost–Probability Relationship ΔC = incremental reconstruction cost; C = total failure trajectory cost; ΔP = absolute reduction in failure probability

Result	Interpretation	Clinical Meaning
ΔC < C × ΔP	Reconstruction economically justified	Expected value supports reconstruction
ΔC = C × ΔP	Indeterminate (requires clinical judgment)	Additional clinical judgment determines strategy
ΔC > C × ΔP	Reconstruction not economically justified	Reconstruction not justified on economic grounds alone

The Tier 1 / low-ΔP quadrant appropriately shows thresholds below $50,000, confirming the model’s bidirectionality: in low-risk environments with modest risk reduction, routine closure may be the economically appropriate strategy.

These threshold relationships are independent of reimbursement structure and reflect underlying cost dynamics rather than pricing assumptions. The decision boundary shifts with clinical inputs, not contractual context. All cost values referenced in this section represent illustrative, literature-derived estimates used for modeling purposes and do not reflect empirically measured costs.

Methodological symmetry: CASCADE is not a justification framework for reconstruction. It is a selection framework that may favor either strategy depending on risk conditions. In lower-risk environments, CASCADE analysis will frequently support conservative management. This bidirectionality is essential to the model’s credibility and defensibility.

Worked Example: Low-Risk Wound - Routine Closure Favored

The following example applies CASCADE analysis to a low-risk wound environment to demonstrate the model’s bidirectional output (Tables 9, 10). The purpose is to confirm that CASCADE does not predetermine in favor of reconstruction across all clinical presentations.

**Table 9:** Step 1 – Baseline Clinical Risk Assessment (CRS) Example for Wound Failure CRS = Clinical Risk Score. Step 1 establishes baseline failure risk using pre-intervention patient and wound characteristics. Scores reflect the presence or absence of defined risk factors prior to any reconstructive decision and are used to assign an initial risk tier (Table 3), which informs downstream probability and cost modeling.

Risk Factor	Points
No hardware present	0
No perfusion compromise	0
Tension-free closure achievable	0
No significant soft tissue deficit	0
Comorbidity: controlled diabetes	1
Cumulative CRS =	1 → LOW RISK

**Table 10:** Steps 2–5 – Probability Assignment, Cost Modeling, Threshold Calculation, and Decision Analysis (Worked Example) P₁ = baseline failure probability with routine closure; P₂ = failure probability with reconstruction; ΔP = absolute risk reduction. C = total failure trajectory cost (Table 5). ΔP × C defines the maximum economically justified incremental reconstruction cost (threshold). Step 5 compares ΔC to ΔP × C to determine whether reconstruction is economically favored.

Variable	Value/Rationale
Step 2: P₁ (routine closure)	0.07 (Low risk tier midpoint)
Step 2: P₂ (reconstruction)	0.04 (Low risk tier midpoint)
Step 2: ΔP	0.03
Step 3: Failure Cost Tier (FCT Selection)	Tier 1 — Limited: SSI risk without hardware or complex anatomy, short LOS extension expected if failure
Step 3: C (midpoint)	175000
Step 4: ΔP × C (threshold)	ΔP × C = 0.03 × $175,000 = $5,250
Step 5: Decision (ΔC compared to ΔP × C threshold)	Reconstruction is economically favored only if ΔC < $5,250. In practice, reconstructive costs exceed this threshold in nearly all cases; therefore, CASCADE analysis does not support reconstruction in this low-risk scenario on expected-value grounds. Clinical decision-making may still support reconstruction based on non-economic factors.

Bidirectionality confirmed: In this low-risk environment, CASCADE analysis supports routine closure as the economically rational strategy. The decision threshold of $5,250 is below the incremental cost of advanced reconstruction in virtually all institutional contexts. This output is intentional and reflects the model operating correctly: CASCADE identifies when reconstruction does not improve the expected total episode cost, not only when it does.

Worked Example: High-Risk Posterior Spine Wound

The following applies CASCADE analysis to a representative high-risk case to demonstrate the decision algorithm under realistic clinical inputs (Tables 11, 12).

**Table 11:** Step 1 – Baseline Clinical Risk Assessment (CRS) Example for High-Risk Wound Scenario CRS = Clinical Risk Score. Step 1 establishes baseline failure risk using pre-intervention patient and wound characteristics. This example demonstrates a high-risk composite profile with multiple additive risk factors, resulting in classification into a high-risk tier (Table 3), which is associated with increased failure probability and downstream cost burden in the CASCADE model.

Risk Factor	Points
Hardware: spinal instrumentation	2
Perfusion compromise: compromised soft tissue perfusion (thin flaps)	2
Soft tissue destruction: dead space	2
Operative factors: prolonged operative time	1
Comorbidity: diabetes, obesity	1
Cumulative CRS	8 (High Risk Tier)

**Table 12:** Steps 2–5 — Probability Assignment, Failure Cost Modeling, Threshold Calculation, and Decision Analysis (High-Risk Scenario) P₁ = baseline failure probability with routine closure; P₂ = failure probability with reconstruction; ΔP = absolute risk reduction. C = total failure trajectory cost (Table 5). ΔP × C defines the maximum economically justified incremental reconstruction cost (threshold). ΔC represents the actual incremental cost of reconstruction. Reconstruction is economically justified when ΔC < ΔP × C.

Variable	Value/Rationale
P₁ (routine closure)	0.20 (High-risk tier midpoint)
P₂ (reconstruction)	0.07 (high-risk tier midpoint)
ΔP	0.13
Failure Cost Tier (FCT selection)	Tier 3 (Severe failure trajectory): hardware exposure, complex anatomy, anticipated multi-stage failure progression
C (Failure trajectory cost, midpoint)	$600,000
ΔP × C (threshold)	0.13 × $600,000 = $78,000
Sensitivity analysis (higher cost scenario, C = $800K)	0.13 × $800,000 = $104,000
ΔC (incremental reconstruction cost)	Case-specific; externally determined
Decision	Reconstruction is economically justified when ΔC < ΔP × C ($78,000–$104,000 depending on cost assumptions)

Clinical context: Failure in this setting typically progresses from wound dehiscence to hardware exposure, necessitating urgent return to the operating room for irrigation and debridement. Hardware exposure in the posterior spine introduces infection risk, requiring prolonged intravenous antibiotics, extended inpatient stay, and, in many cases, staged reconstruction with regional or free-tissue flap coverage. Each stage occurs in a progressively more hostile wound environment - scarred, infected, and mechanically compromised - increasing the complexity and cost of subsequent intervention. This is the documented natural history of posterior spine wound failure in high-risk patients [5,6].

Decision consequence: Under these conditions, the expected failure liability (ΔP × C) ranges from $78,000 to $104,000 across the evaluated failure cost range. Across the majority of scenarios at this risk tier, this threshold exceeds the incremental cost of reconstruction, indicating that selection of the lower-cost initial strategy may produce a higher expected total episode cost under a trajectory-based evaluation framework. Directional conclusions remain consistent across bounded input ranges; specific estimates vary with case-level inputs.

Illustrative Case Contrast

The following scenarios illustrate the potential cost difference between trajectory types (Table 13). These are constructed illustrative examples, not matched clinical data or observed case-level measurements. Cost values are literature-informed estimates used to demonstrate model behavior; they should not be interpreted as empirical findings.

**Table 13:** Comparative Clinical Trajectories — Early Definitive Reconstruction vs. Failure-Driven Care Pathway This table illustrates representative clinical trajectories following early definitive reconstruction versus failure-driven care. Cost estimates align with failure trajectory ranges presented in Table 5.

Domain	Patient A (Early Definitive Reconstruction)	Patient B (Failure Trajectory)
Initial Strategy	High-risk wound identified at index procedure	Primary closure under tension
Index Intervention	Immediate debridement and flap coverage	Primary closure without definitive reconstruction
Early Course	No complications	Postoperative day 7: wound dehiscence with infection
Subsequent Interventions	None	Multiple reoperations (washout, VAC therapy, delayed flap)
Resource Utilization	Short inpatient stay	Prolonged inpatient course with SNF placement and home health utilization
Readmissions	None	Multiple readmissions
Total Episode Cost	$120,000	$600,000–$700,000 (estimated)

Illustrative cost differential: approximately $560,000. This estimate is presented only to demonstrate how CASCADE behaves when two constructed scenarios are compared using the model’s assumptions. The figure is not derived from matched cohort data and should be interpreted as an illustrative model output rather than an observed empirical result.

Population-Level Modeling: Cohort Implications

To illustrate aggregate model implications, CASCADE inputs were applied to a hypothetical 100-case cohort of high-risk wounds using the parameter ranges documented in this framework (Table 14). This is a constructed modeling exercise, not a real-world cohort study. The arithmetic converts literature-informed complication-rate reductions into estimated avoided cost exposure (Table 15) [1,3,4].

**Table 14:** Estimated Failure Event Reduction With Early Reconstruction (Per 100 Cases) ΔP represents absolute risk reduction in failure events with early reconstruction compared to routine closure. Values reflect representative ranges derived from high-risk wound populations and correspond to probability differences used in the CASCADE model.

Scenario	Complication Rate	Failure Events per 100 Cases
Routine closure (baseline)	15–25%	15–25
Early reconstructive strategy	5–10%	5–10
Absolute reduction (ΔP)	-	10–20 fewer events

**Table 15:** Estimated Avoided Cost Exposure With Early Reconstruction (Per 100-Case Cohort) Avoided cost exposure is derived from estimated reductions in failure events (Table 14) multiplied by representative failure trajectory costs (Table 5). Values are presented for a 100-case cohort and normalized per case to illustrate the expected economic impact at the patient level.

Bound	Calculation	Total Avoided Cost Exposure (USD)
Lower bound	10 failures avoided × $400,000	$4,000,000
Upper bound	20 failures avoided × $1,000,000	$20,000,000
Normalized per case	Distributed across a 100-case cohort	$40,000–$200,000 per case

Model sensitivity: Even at conservative illustrative inputs (10% absolute risk reduction and a Tier 1 failure cost floor of $100,000), the model generates an estimated threshold of 0.10 × $100,000 = $10,000 per case. Across a hypothetical 100-case cohort with 10 avoided failure events, the estimated avoided expenditure would reach $1,000,000. These values are illustrative and depend on the selected probability and cost assumptions. The analysis supports directional interpretation but does not replace validation using observed datasets.

Discussion

Economic Unit of Analysis

In multi-stage clinical pathways, the relevant economic unit is the total episode cost conditional on strategy selection, not the cost of the index procedure evaluated in isolation. Because downstream events are conditional on the operative decision, they cannot be separated from it for purposes of economic evaluation.

Evaluation limited to the index procedure excludes the downstream events that are themselves determined by that decision. As a result, procedural-only evaluation does not merely underestimate cost - it evaluates a different economic unit than the one placed at risk. This distinction is not a matter of degree; it is a structural difference in what is being measured.

A model that excludes trajectory-dependent cost evaluates a different economic unit and cannot determine total episode value. CASCADE corrects this by defining the economic unit as the expected total episode cost conditional on strategy selection at the index operation. Under this definition, the cost of a reconstructive decision is not the price of the procedure performed; it is the probability-weighted sum of all downstream events that the decision initiates or forecloses.

Staged Reconstruction as Risk Containment

Staged reconstruction is a clinical strategy designed to interrupt the failure trajectory before it begins by addressing the biological and mechanical drivers of wound breakdown. This approach is consistent with orthoplastic principles and supported by evidence demonstrating improved outcomes with early definitive soft tissue coverage in high-risk wounds [9-16].

The perceived cost savings of routine closure in high-risk wounds represent cost deferral rather than cost reduction: the index procedure carries a lower immediate charge, but the aggregate episode cost increases substantially when failure occurs. This pattern has been consistently demonstrated in trauma and reconstructive literature evaluating delayed versus early soft tissue management [5-7,14-16].

The four-phase reconstructive model includes (a) debridement: elimination of non-viable, contaminated, or ischemic tissue to establish a clean wound bed capable of supporting reconstruction; (b) optimization: glycemic control, nutritional repletion, vascular assessment, and antibiotic stewardship prior to definitive closure; (c) reconstruction: vascularized tissue coverage with tension-free closure to restore structural integrity and perfusion; and (d) recovery: a defined postoperative monitoring protocol with clear endpoints and discharge criteria.

Literature Basis for Complication-Rate Reduction

Published studies across high-risk reconstructive settings demonstrate substantial reductions in wound complication rates with appropriately selected early reconstructive strategies, commonly in the range of 30-60% in selected cohorts [5-8,14-16]. Higher effect sizes have been reported in specific high-risk settings, including early orthoplastic trauma reconstruction and flap-based oncologic closure [9-13]. These findings are supported by systematic reviews and consensus recommendations emphasizing the importance of timing and definitive coverage in reducing complication rates [14-16]. Table 16 outlines these findings, including reported baseline failure rates, outcomes following early reconstruction, and corresponding relative risk reductions across representative clinical scenarios.

**Table 16:** Reported Failure Rates With and Without Early Reconstruction Across High-Risk Clinical Settings Failure rates represent literature-reported ranges across high-risk wound settings. Relative risk reduction reflects proportional decreases in failure with early definitive reconstruction compared to routine closure. Values are used to inform probability inputs (ΔP) within the CASCADE model.

Setting	Baseline Failure Rate	Failure Rate With Early Reconstruction	Relative Risk Reduction (%)	Citations
High-risk posterior spine	20–30%	8–20%	30–60%	[5,6]
Trauma (open fractures/soft tissue loss)	25–50%	10–25%	40–60%	[9-11,14-16]
Oncologic resection (perineal/pelvic)	30–50%	15–30%	40–60%+	[12,13]
Diabetic/immunocompromised	20–40%	15–25%	25–50% (heterogeneous populations)	[7,8]

System-Level Cost Amplifiers and Institutional Liability

The central implication is that failure-related cost is not confined to the operative encounter; it may extend into institutional performance domains that are typically invisible in index-procedure accounting.

Failure trajectories generate costs that extend beyond direct episode expenditure into institutional performance domains. Table 17 summarizes these downstream cost domains, including mechanisms of cost generation and their associated financial impact. These costs are structurally excluded from per-case accounting but are nonetheless real financial consequences of the index operative decision. When evaluated at the service-line level, they convert individual wound failures into aggregate institutional liabilities [3,8,17].

**Table 17:** Institutional Financial Penalties Associated With Failure Trajectories Not Captured in Index Episode Costing These institutional cost domains represent downstream financial effects of failure trajectories that are not captured in per-case procedural reimbursement or index episode cost accounting. They may contribute to incomplete estimation of the economic burden of reconstructive failure.

Institutional Penalty Domain	Mechanism of Cost Generation	Downstream Financial Impact
CMS Readmission Penalties	Value-based penalties linked to 30-day readmission rates	Direct financial penalties per excess readmission; reduced hospital margin and quality scores
Bundled Payment Exposure	Unplanned utilization exceeds bundled payment thresholds	Unreimbursed costs above bundle cap; negative contribution margin
OR Throughput Disruption	Unplanned reoperations displace scheduled higher-margin cases	Lost elective case revenue; associated opportunity cost
Quality Metric Deterioration	Complication rates adversely affect payer contracts and referral patterns	Long-term case volume reduction, payer mix degradation, and reimbursement pressure

Across a service line with meaningful surgical volume, failure rates in high-risk wounds may create substantial institutional cost exposure. When the failure rate in a defined high-risk population is 15-25%, and the expected cost of each failure falls within published ranges, aggregate exposure can be estimated using expected-value methods. For example, at 100 cases per year with a 20% failure rate and an estimated $400,000-$600,000 average failure cost, annual modeled exposure would range from $8,000,000 to $12,000,000. This is an illustrative expected-value calculation based on published parameters, not an observed institutional dataset [1,3,4,17].

Single-point procedural pricing metrics, including median contracted-rate benchmarks such as the Qualifying Payment Amount (QPA), evaluate index procedural pricing across broad populations that may include low-complexity cases, elective procedures, and patients without the risk profile that initiates failure trajectory entry. Such metrics do not directly incorporate probability-weighted downstream expenditure, a limitation consistent with the broader value-based healthcare literature, which emphasizes total episode cost and downstream resource utilization [18,19].

CASCADE evaluates a different economic unit: expected total episode expenditure conditional on strategy selection in a risk-stratified wound environment. Differences between procedural pricing metrics and CASCADE outputs, therefore, reflect differences in the unit of analysis rather than differences in pricing assumptions. In high-risk reconstructive settings, these measures should not be interpreted as interchangeable.

Generalizability and Implementation Challenges

CASCADE is intended for high-risk, resource-intensive wound environments and may not generalize to low-risk wounds, uncomplicated elective closures, or settings in which reliable tension-free closure is achievable without advanced reconstruction. Application across different healthcare systems may also vary because facility cost structures, post-acute utilization, bundled payment exposure, and payer-specific reimbursement rules differ substantially by institution and geography. Practical implementation requires consistent documentation of CRS drivers, transparent assignment of FCT, and access to reasonable estimates of incremental reconstruction cost. These requirements may limit immediate use in settings without a mature cost-accounting infrastructure.

Clinical Decision Elements and Documentation Requirements

CASCADE analysis generates specific clinical documentation requirements corresponding to each element of the reconstructive decision (Table 18). Three elements should be independently documented to establish the clinical basis for reconstructive strategy selection within a trajectory-based framework.

**Table 18:** Clinical Criteria Establishing Indications for Definitive Reconstructive Closure These criteria define clinical conditions under which routine closure is mechanically or biologically unsound. Their presence supports the necessity of definitive reconstructive strategies and informs risk stratification within the CASCADE model.

Clinical Requirement	Objective Clinical Basis
Debridement Requirement	Presence of non-viable, contaminated, or ischemic tissue that cannot be incorporated into a stable closure; establishes that routine closure is contraindicated
Vascularized Coverage Requirement	Inadequate perfusion to support primary intention healing (e.g., exposed hardware, irradiated tissue, peripheral vascular disease, poorly controlled diabetes, significant dead space)
Tension-Free Closure Requirement	Closure cannot be achieved without pathologic tension; tension is a primary mechanical driver of dehiscence, particularly in high-motion anatomical regions, requiring tissue advancement, rotation, or transfer

These elements represent the clinical predicates linking operative decision-making to failure probability reduction. Independent documentation of each establishes the medical necessity of reconstruction within a trajectory-based framework and connects the operative record directly to the CASCADE decision analysis. This approach is consistent with contemporary surgical frameworks that emphasize early, definitive reconstruction, risk stratification, and consensus-based decision-making in complex wounds [20].

Case-Level Application

In case-level evaluation, the relevant question is whether the selected reconstructive strategy reduced expected total episode cost relative to the available alternative in the documented risk environment. This determination is made using documented risk factors, the expected failure trajectory given wound characteristics and anatomy, and the anticipated effect of the selected reconstructive approach on failure probability.

The determination does not require institution-specific billing data. It relies on three estimable inputs (CRS-derived failure probability, FCT-defined trajectory cost, and case-level incremental reconstruction cost), each bounded within published ranges incorporated into this framework (Table 19) [1-7,14-20].

**Table 19:** Required Documentation Elements Linking Clinical Decision-Making to CASCADE Model Inputs These documentation elements establish a direct linkage between clinical decision-making and model inputs within the CASCADE framework. Their presence supports transparent justification of risk stratification, probability assignment, cost modeling, and expected-value calculations in reconstructive decision analysis.

Required Documentation Element	CASCADE Model Function
CRS drivers documented	Defines baseline risk tier and assigns failure probability inputs (P₁, P₂, ΔP)
Expected failure trajectory documented	Defines failure cost tier (FCT) and associated total episode cost range (C)
Reason routine closure is inadequate	Establishes clinical necessity for reconstruction (debridement, vascularized coverage, tension-free closure requirements)
Reconstructive mechanism selected	Defines incremental intervention (ΔC) and confirms strategy (e.g., flap, graft, staged reconstruction)
Anticipated prevented downstream events	Links intervention to reduction in failure trajectory and expected total episode cost differential

Limitations

CASCADE is a conceptual, literature-derived decision framework rather than a primary data analysis, claims-based economic study, or statistically validated predictive model. Its inputs have not been prospectively or retrospectively validated against institutional cost-accounting datasets, NSQIP-linked complication data, Medicare claims, or state all-payer databases. Formal validation using such datasets represents a necessary next step.

The CRS is a nonvalidated clinical stratification tool. Its point assignments are expert-informed and literature-supported but are not statistically derived. The model uses additive scoring and does not account for interaction effects between risk factors, such as the combined influence of diabetes, contamination, perfusion compromise, and hardware exposure. Future work should test CRS weighting using regression-based methods, Delphi consensus, or other structured validation approaches.

Cost inputs are bounded, literature-informed estimates rather than inflation-adjusted, geography-specific, or institution-specific measurements. Actual costs may vary by payer mix, hospital cost structure, year, region, post-acute utilization, and case complexity. The sensitivity analysis is deterministic and uses representative values rather than probabilistic sampling across full cost distributions. Future studies should evaluate lower- and upper-bound thresholds and may incorporate Monte Carlo simulation.

The illustrative patient scenarios and hypothetical 100-case cohort are constructed examples intended to demonstrate model behavior. They are not matched cohort data and should not be interpreted as observed empirical outcomes. Effect-size estimates for complication reduction reflect heterogeneous clinical literature and vary by indication, wound environment, timing of reconstruction, patient risk profile, and reconstructive modality.

CASCADE is intended for high-risk wound environments where failure trajectory entry is clinically meaningful. It is not designed for universal application to all wounds. In low-risk cases, routine closure may remain clinically and economically appropriate. Finally, CASCADE evaluates cost relationships and trajectory-based decision-making; it does not define reimbursement levels or procedural pricing standards.

## Conclusions

Complex wound reconstruction is best evaluated not only by the index procedure performed but also by the clinical trajectory that may follow. CASCADE provides a conceptual, literature-derived framework for evaluating reconstructive strategies through probability-weighted cost analysis, using three estimable parameters: incremental reconstruction cost, absolute risk reduction, and failure trajectory cost. The model is bidirectional and may support either routine closure or definitive reconstruction depending on the documented risk environment.

Within high-risk wound settings, CASCADE helps make downstream episode-level consequences visible at the point of reconstructive decision-making. The framework does not predict exact patient outcomes, does not determine reimbursement levels, and has not yet been validated against observed institutional or claims-based datasets. Its primary value is to provide a transparent structure for evaluating treatment trajectories under uncertainty and to support future empirical validation in complex wound reconstruction.
